# Rab27A Regulates Transport of Cell Surface Receptors Modulating Multinucleation and Lysosome-Related Organelles in Osteoclasts

**DOI:** 10.1038/srep09620

**Published:** 2015-04-16

**Authors:** Megumi Shimada-Sugawara, Eiko Sakai, Kuniaki Okamoto, Mitsunori Fukuda, Tetsuro Izumi, Noriaki Yoshida, Takayuki Tsukuba

**Affiliations:** 1 Division of Dental Pharmacology, Graduate School of Biomedical Sciences, Nagasaki University, Nagasaki 852-8588, Japan; 2 Division of Orthodontics and Dentofacial Orthopedics, Graduate School of Biomedical Sciences, Nagasaki University, Nagasaki 852-8588, Japan; 3 Laboratory of Membrane Trafficking Mechanisms, Department of Developmental Biology and Neurosciences, Graduate School of Life Sciences, Tohoku University, Aobayama, Aoba-ku, Sendai, Miyagi 980-8578, Japan; 4 Department of Molecular Medicine, Institute for Molecular and Cellular Regulation, Gunma University, Maebashi 371-8512, Japan

## Abstract

Rab27A regulates transport of lysosome-related organelles (LROs) and release of secretory granules in various types of cells. Here, we identified up-regulation of Rab27A during differentiation of osteoclasts (OCLs) from bone-marrow macrophages (BMMs), by DNA microarray analysis. Rab27A deficiency in OCLs, using small interfering RNA (siRNA) knockdown in RAW-D cell line or BMMs derived from *ashen* mice, which display genetic defects in Rab27A expression, induced multinucleated and giant cells. Upon stimulation with macrophage-colony stimulating factor (M-CSF) and receptor activator of nuclear factor kappa-B ligand (RANKL), essential cytokines for OCL differentiation, phosphorylation levels of extracellular signal-regulated kinase (Erk), proto-oncogene tyrosine-protein kinase (Src), and p-38 were slightly enhanced in *ashen* BMMs than in wild-type BMMs. The cell surface level of c-fms, an M-CSF receptor, was slightly higher in *ashen* BMMs than in wild-type BMMs, and down-regulation of RANK, a RANKL receptor, was delayed. In addition to receptors, OCLs derived from *ashen* mice exhibited aberrant actin ring formation, abnormal subcellular localization of lysosome-associated membrane protein (LAMP2) and cathepsin K (CTSK), and marked reduction in resorbing activity. Thus, these findings suggest that Rab27A regulates normal transport of cell surface receptors modulating multinucleation and LROs in OCLs.

The Rab family of small GTPases mediates membrane trafficking events such as vesicle formation, vesicle movement and membrane fusion^1^. A member of this family, Rab27A, has been extensively studied^2^. Griscelli syndrome type 2 is a genetic disorder affecting Rab27A in humans, and it is characterized by hypopigmentation of the skin and eyes, immunodeficiency^3^,^4^. Rab27A is widely expressed on secretory granules in various secretory cells, such as endocrine and exocrine cells and various leukocytes^5^. Rab27A is particularly involved in regulation of transport of “lysosome-related organelles (LROs)”^6^,^7^. LROs resemble morphologically lysosomes with electron-dense protein deposits, and contain most lysosomal proteins, and have a low luminal pH. However, they display many distinct morphological, functional, and compositional characteristics^8^. LROs include the melanosomes in melanocytes^9^, lytic granules in lymphocytes^3^,^10^, delta granules in platelets^11^, and “secretory” lysosomes in osteoclasts (OCLs)^12^. Compared to the other LROs, the transport mechanisms of LROs in OCLs are not well known.

OCLs are multinucleated giant cells that mainly participate in bone resorption^13^,^14^. OCLs are formed by the fusion of mononuclear progenitors of the monocyte/macrophage lineage^15^. When OCLs undergo bone resorption, they form a specialized LRO, termed as the “ruffled border”^16^,^17^. The ruffled border generates an acidic extracellular microenvironment called as resorption lacuna, which is surrounded by an actin ring^18^. Several acid hydrolases are secreted in the resorption lacuna, which is formed on the bone surface^19^. Among these acid hydrolases, cathepsin K (CTSK) and tartrate-resistant acid phosphatase (TRAP, also known as ACP5) are highly expressed and secreted by differentiated OCLs. CTSK is a lysosomal cysteine protease that degrades type I collagen, the major component of bone matrix^20^,^21^,^22^. TRAP is a metallo-phosphoesterase involved in bone matrix degradation, and removal of mannose 6-phosphate (Man-6-P) residues from acid hydrolases^23^,^24^. However, the detailed transport mechanisms of LROs in OCLs are yet to be elucidated. In addition, the extent of involvement of Rab27A in the transport of LROs in OCLs is still unclear.

In this study, we identified the up-regulation of Rab27A during OCL differentiation from bone-marrow macrophages (BMMs), using DNA microarray analysis. Moreover, we have demonstrated, by knock-down using small interfering RNA (siRNA) and the Rab27A-deficient *ashen* mice, that Rab27A regulates the transport of LROs and cell surface receptors, thereby modulating the cell size in OCLs.

## Results

### Expression of Rab27A increases during OCL differentiation

To identify a gene which regulates membrane trafficking during OCL differentiation, we performed DNA microarray analysis. Total RNA was obtained from BMMs treated with M-CSF (30 ng/ml) and RANKL (50 ng/ml), and cultured for 72 h on a plastic surface or a dentin slice. We observed that the OCLs cultured on the plastic surface were differentiated rapidly into OCL rather than on the dentin slice. Therefore, we compared the mRNA levels of OCLs cultured under the two different conditions. Of the total of 40,130 genes identified during the analysis, 1,363 were up-regulated and 881 genes were down-regulated. Indeed, OCL marker genes such as calcitonin receptor (CTR), cathepsin K (CTSK), transmembrane 7 superfamily member 4 (DC-STAMP), carbonic anhydrase 2, and TRAP were up-regulated (Supplementary Figure S1A). Among the several up-regulated genes, we focused on Rab27A, since Rab27A expression showed an increased, but that of Rab27B decreased during OCL differentiation.

We further measured the mRNA levels of Rab27A and Rab27B in MC3T3-E1 cells (murine osteoblastic precursor cell line), RAW-D cells (sub-clone of the murine macrophage cell line RAW264.7, which has a high capacity for differentiation into OCLs)^25^, and RANKL-stimulated RAW-D cells (OCLs). Quantitative real-time PCR analysis showed that the mRNA level of Rab27A in RAW-D cells was a 4.5-fold higher than that in MC3TC-E1 cells (Supplementary Figure S1B). Moreover, upon stimulation with RANKL, the Rab27A mRNA expression in mature OCLs was significantly increased compared to that in unstimulated RAW-D cells (Supplementary Figure S1B). However, the mRNA levels of Rab27B in RAW-D and OCLs were not detectable (Supplementary Figure S1B). Thus, we concluded that Rab27A expression was significantly increased during differentiation from macrophages into OCLs.

### Rab27A is localized in lysosomes in RAW-D cells

We next determined the subcellular localization of Rab27A in RAW-D cells. Since the staining pattern of endogenous Rab27A in the OCL precursor cell RAW-D and RANKL-stimulated OCLs differentiated from RAW-D cells, with several anti-Rab27A antibodies was obscure, we analyzed overexpression of Rab27A-Green Fluorescent Protein (GFP) in RAW-D cells. We compared the localization of Rab27A against several organelle marker proteins. As shown in Supplementary Figure S2, the Rab27A-GFP protein was extensively colocalized with LAMP2 (late endosomes/lysosomes), but not with GM130 (Golgi) and calnexin (endoplasmic reticulum; ER). These results indicate that Rab27A is localized in the lysosomes and late endosomes in RAW-D cells.

### Knockdown of Rab27A causes formation of multinucleated, giant OCLs

**Figure 1:**
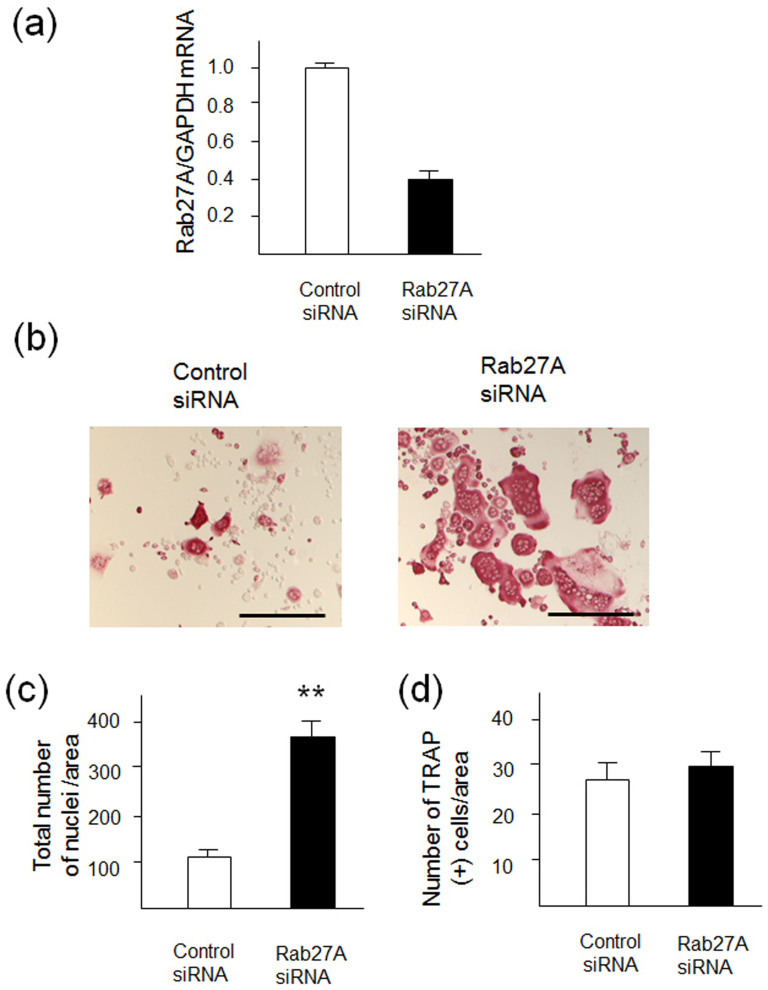
Knockdown of Rab27A in RAW-D derived OCLs after stimulation with RANKL. (a) Knockdown efficiency was measured by mRNA levels of Rab27A. After incubation with 10 p mol siRNA for 24 h, cells were cultured for additional for 5 days in the presence of RANKL (50ng/mL). (b) RAW-D cells were transfected with either a control siRNA or a siRNA specific for Rab27A. After stimulation with RANKL (50 ng/mL) for 72 h, OCLs were analyzed by TRAP-staining. (c) Total nucleus number of TRAP positive multinucleated OCLs, but not TRAP negative mononucleated cells following a 72 h culture, was counted per viewing field. ** *P* < 0.01. (D) The number of TRAP-positive multinucleated OCLs per viewing field was counted. Bar: 50 μm.

To explore the role of Rab27A during OCL differentiation, we performed knockdown by siRNA transfection in RANKL-stimulated RAW-D cells. We determined the knockdown efficiency of Rab27A in the RAW-D derived OCLs (Fig. 1A). Rab27A siRNA potently inhibited its expression level down to approximately 40% as compared to that obtained from a control siRNA (Fig. 1a). Under these conditions, Rab27A depletion induced considerably distinct morphological characteristics in OCLs such as multinucleated and giant cells, when both the cells were cultured for 72 h after stimulation with RANKL (Fig. 1b). A total nucleus number count in TRAP-positive multinucleated cells transfected with the control siRNA and Rab27A siRNA showed a higher nucleus number in Rab27A depleted OCLs, compared to that seen in the control OCLs (Fig. 1c). This suggests that the control cells largely remained as TRAP-negative mononuclear cells (Fig. 1c). However, the number of TRAP positive multinucleated OCLs was not different between the control and Rab27A knockdown OCLs (Fig. 1d). These results indicate that Rab27A depletion in OCLs induce multinucleation and larger cell formation.

### OCLs derived from Rab27A-deficient (*ashen*) mice displays multinucleation and giant cell formation

**Figure 2:**
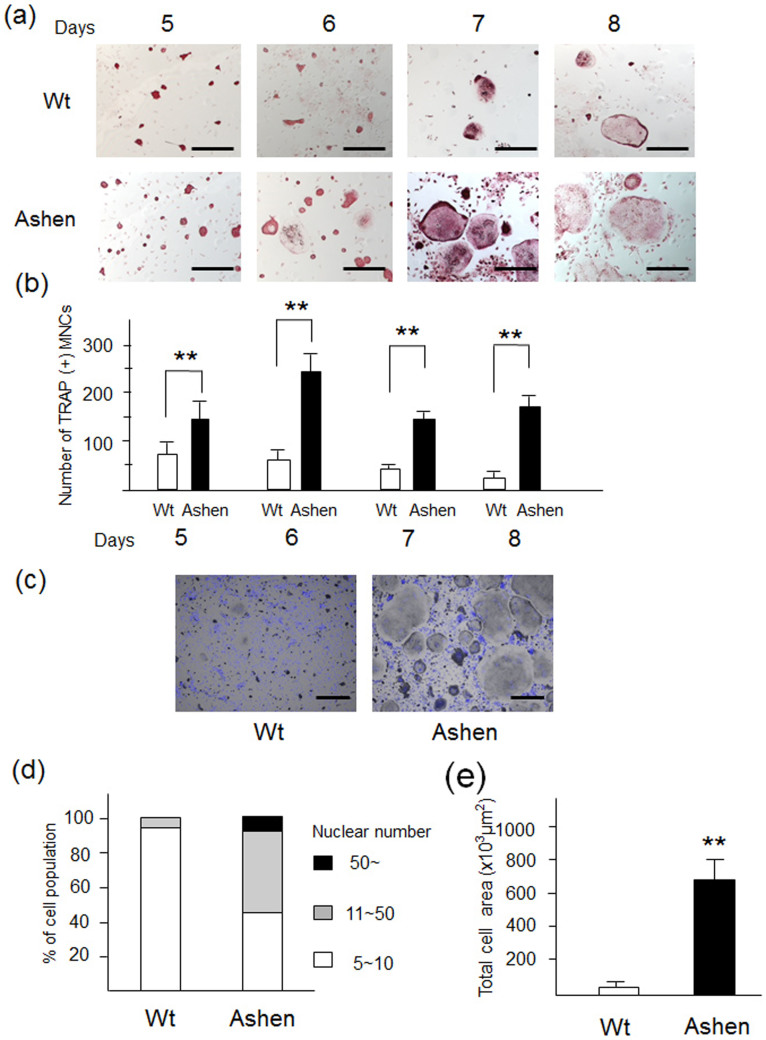
Phenotypes of OCLs derived from control mice (wild-type) and *ashen* mice. (a) BMMs derived from wild-type and *ashen* mice were cultured with M-CSF (10 ng/ml) and RANKL (50 ng/ml) for the indicated days. The cells were fixed and stained for TRAP. Bar: 50 μm. (b) The number of TRAP-positive multinucleated cells was counted at each indicated day. (c) The cells were fixed and stained with nuclear staining DAPI. Bar: 50 μm. (d) the nuclear numbers were counted and classified as 5 ~ 10 (*white bar*), 11 ~ 50 (*gray bar*), or more than 50 (*black bar*). (e) The individual cell area was analyzed by using the BZ analyzer software, dynamic cell count system (Keyence).

Given the importance of Rab27A in osteoclastogenesis, we compared the phenotypes of OCL differentiation in BMMs derived from *ashen* mice, which show genetic defects in Rab27A expression, and control mice^10^,^26^. The BMMs were cultured with media containing M-CSF and RANKL, essential cytokines for OCL maturation, for 5, 6, 7, and 8 days. TRAP staining revealed that OCLs from *ashen* mice exhibited a markedly bigger size compared to OCLs from the wild-type mice (Fig. 2a). The number of TRAP-positive cells was higher in *ashen* mice than that of the wild-type mice at all the indicated days (Fig. 2b). Further, we counted the nuclear number of the OCLs following nuclear staining with DAPI (Fig. 2c). The OCLs from *ashen* mice containing more than 11 nuclei accounted for approximately 50% of the total number, whereas those from the wild-type did for only approximately 10% (Fig. 2d). A calculation of the cell area of OCLs from wild-type and *ashen* mice at 8 days showed a higher cell area in *ashen* mice than in wild-type mice (Fig. 2e).

### Partially enhanced maturation of *ashen* OCL

**Figure 3:**
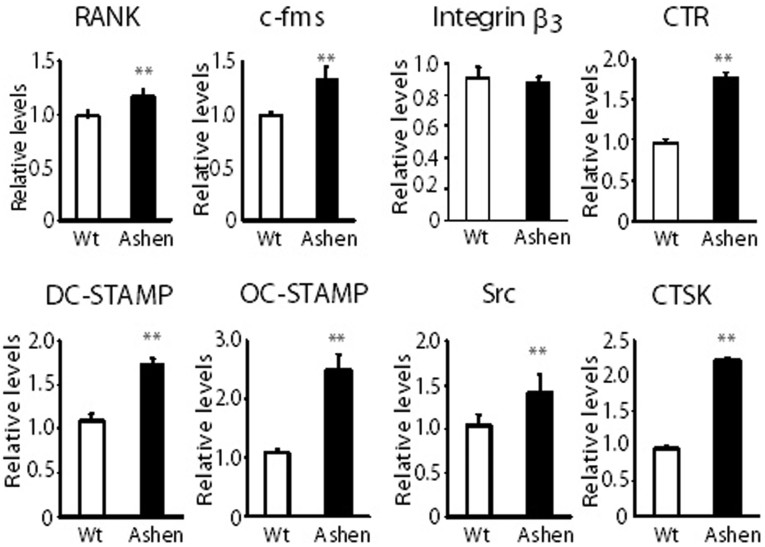
Comparison of mRNA levels of various marker proteins OCLs from wild-type and *ashen* mice. BMMs derived from wild-type and *ashen* mice were cultured with M-CSF (10 ng/ml) and RANKL (50 ng/ml) for 5 days. After isolation of mRNA in these cells, real-time PCR was performed. **P < 0.01, *P < 0.05 for the indicated comparisons.

To examine the differences between wild-type and *ashen* OCLs, we compared mRNA levels of various OCL marker genes in wild-type and *ashen* OCLs. As shown in Fig. 3, quantitative real-time PCR revealed that mRNA levels of RANK, c-fms, CTR, DC-STAMP, OC-STAMP, and CTSK were significantly higher in *ashen* OCLs compared to wild-type OCLs. However, the levels of integrin β3 and Src were indistinguishable in wild-type and *ashen* OCLs. (Fig. 3). These results indicate the partially enhancement of marker gene expression in *ashen* OCLs compared to wild-type OCLs.

### Enhanced signaling of M-CSF and RANKL in OCLs from *ashen* mice

**Figure 4:**
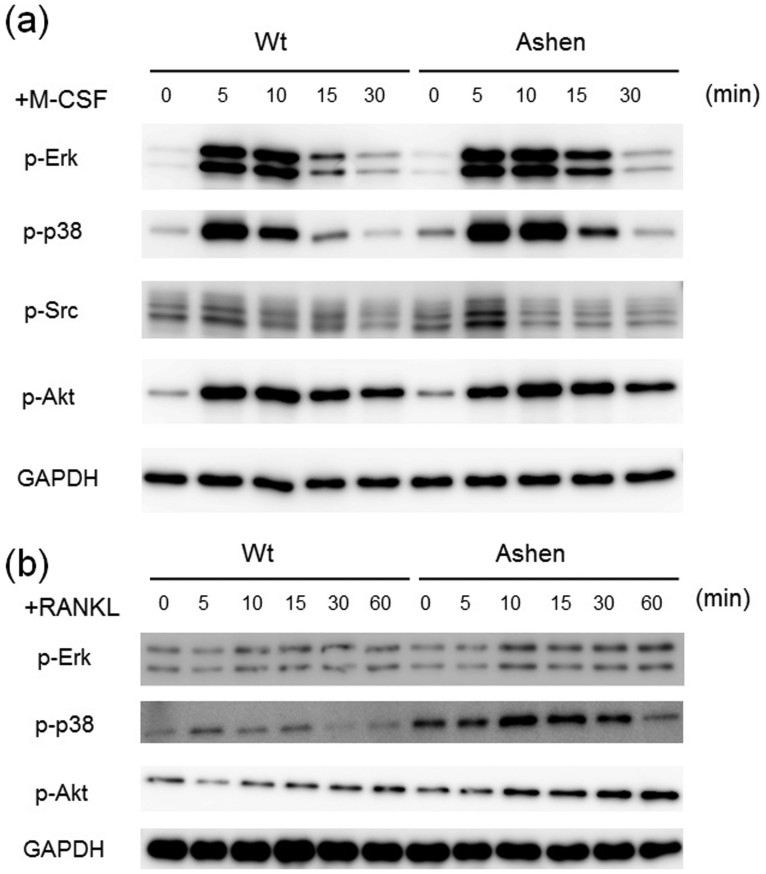
Comparison of signaling levels of OCLs from wild-type and *ashen* mice. (a) BMMs from wild-type and *ashen* mice were pre-incubated for 2 h in serum free media in the absence of M-CSF. After M-CSF addition, the cells were incubated for the indicated times, and consequently harvested. The same protein amounts of cell lysates were subjected to SDS-PAGE followed by western blotting with antibodies against p-Erk, p-p38, p-Src, p-Akt, and GAPDH. (b) BMMs from wild-type and *ashen* mice were pre-incubated for 2 h in serum free media in the absence of RANKL. After adding RANKL, the cells were incubated for the indicated times, consequently harvested. The same protein amounts of cell lysates were subjected to SDS-PAGE followed by western blotting with antibodies to p-Erk, p-p38, p-Akt, and GAPDH.

To investigate molecular mechanisms responsible for larger size of OCLs from *ashen* mice, we tested the signaling levels between wild-type and *ashen* BMMs, which are OCL precursor cells, by stimulation with M-CSF or RANKL. When stimulated with a concentration of M-CSF (10 ng/ml), the phosphorylation levels of Erk, p-38 and Src were slightly enhanced in *ashen* BMMs compared to wild-type cells (Fig. 4a). However, the phosphorylation levels of Akt were indistinguishable between wild-type and *ashen* BMMs (Fig. 4a). Upon stimulation with RANKL (50 ng/ml), the phosphorylation levels of Erk, p-38 and Akt in *ashen* BMMs were increased compared to wild-type cells (Fig. 4b). These results indicate that the enhanced signaling as a result of M-CSF and RANKL stimulation is probably one of the major factors responsible for larger size in *ashen* OCLs than in the wild-type OCLs.

### Increased protein levels of c-fms (MCSF receptor) and RANK (RANKL receptor) in ashen OCLs

**Figure 5:**
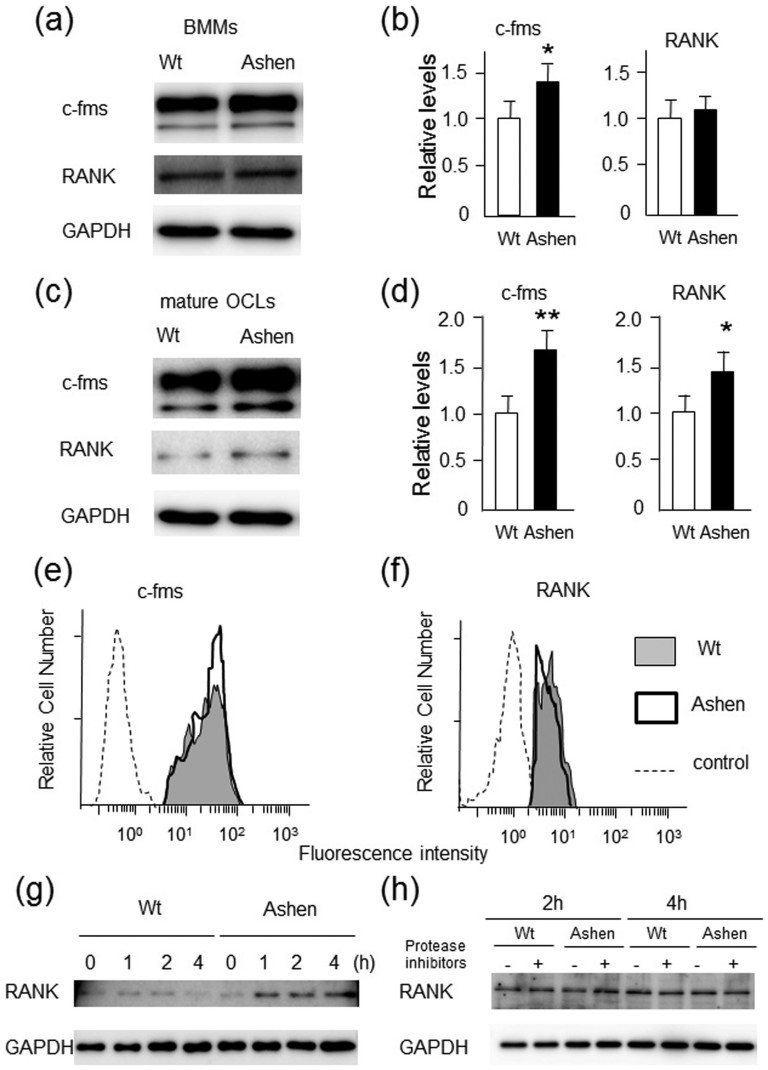
Expression levels of RANK and c-fms in BMMs or OCLs from wild-type and *ashen* mice. BMMs were prepared as per the method described in “Experimental Procedures”. The OCLs were further differentiated from BMMs cultured with RANKL (50 ng/ml) and M-CSF (30 ng/ml) for the 72 h. The same protein amounts of BMM-lysates (a) or OCL-lysates (c) were subjected to SDS-PAGE followed by western blotting with antibodies against RANK, c-fms, and GAPDH. (b, d). Densitometric analysis for the quantification of each protein in the cell lysate of both cell types. The relative levels were defined as the chemiluminescence intensity per mm^2^ measured by LAS4000-mini. The data are represented as the mean ± S.D. of values from four independent experiments. *P < 0.05, **P < 0.01 for the indicated comparison. (e, f) Flow cytometric analysis of surface levels of c-fms (e) or RANK (f) in BMMs. The cells were stained for cell surface with specific antibody against c-fms or RANK, and subsequently stained with a second antibody conjugated with PE. Data are representative of four independent experiments. (g) Down-regulation of RANK in BMMs from wild-type and *ashen* mice. Both the BMMs were treated with RANKL (50 ng/ml), and further incubated at 37°C for the indicated times. The cell lysates were subjected to SDS-PAGE followed by western blotting with antibodies against RANK and GAPDH. (h) Degradation of RANK by lysosomes in BMMs from wild-type and *ashen* mice. Both the BMMs were treated with RANKL (50 ng/ml), and further incubated at 37°C in the absence or presence of E-64 (10 micro g/ml) and pepstain (10 micro g/ml) for the indicated times. The cell lysates were subjected to SDS-PAGE followed by western blotting with antibodies against RANK and GAPDH.

The mechanisms of enhanced signaling in *ashen* OCLs compared to wild-type OCLs were analyzed. First, the total protein levels of c-fms (M-CSF receptor), and RANK (RANKL receptor) in the precursor BMMs and mature OCLs were determined. We observed that the expression level of c-fms in *ashen* BMMs was higher than in wild-type BMMs (Fig. 5a). Densitometric analysis of these proteins also showed that the levels of c-fms in *ashen* BMMs or OCLs were significantly increased as compared to wild-type BMMs (Fig. 5b). However, RANK protein levels between wild-type and *ashen* BMMs were comparable (Fig. 5a, b). Interestingly, during differentiation of BMMs into mature OCLs, the expression levels of c-fms and RANK were significantly up-regulated in *ashen* OCLs compared to those in wild-type OCLs (Fig 5c, d).

We also investigated the surface expression levels of c-fms and RANK by flow cytometory (Fig. 5 e, f). The surface expression of c-fms was slightly higher in *ashen* BMMs than that observed in wild-type BMMs (Fig. 5e). However, the surface expression of RANK in *ashen* BMMs was comparable, but rather slightly lower than that observed in wild-type BMMs (Fig 5f). These results indicate that the enhanced signaling of c-fms in *ashen* cells is probably due to abnormal transport, accumulation and/or slightly increased surface expression levels.

Since we did not determine the mechanisms of the enhanced signaling of RANK in *ashen* BMMs, we further examined the possibility of down-regulation of RANK. Following stimulation with RANKL, the fate of the receptor was determined by western blotting (Fig. 5g). The cellular RANK in wild-type BMMs was gradually degraded for 4 h (Fig. 5g). In contrast, in *ashen* BMMs, the protein levels of RANK were rather increased at approximately 1–4 h (Fig. 5g). To evaluate the effects of lysosomal degradation of RANK, the protein levels of RANK after the ligand stimulation were determined by western blotting in the presence or absence of protease inhibitors such as E-64d, a cysteine protease inhibitor, and pepstatin, an aspartic protease inhibitor (Fig. 5h). However, apparent differences were not observed between in wild-type and *ashen* BMMs (Fig. 5h). These results indicate that Rab27A deficiency caused the partially abnormal down-regulation or transport of RANK, but not abnormal receptor degradation by the lysosomes.

Taken together, we concluded that Rab27A deficiency in OCLs caused the enhanced M-CSF and RANKL signaling through abnormal transport of their respective receptors, c-fms and RANK and/or other adaptor proteins of these receptors.

### Abnormal actin ring formation and altered localization of LAMP2 and CTSK in ashen OCLs

**Figure 6:**
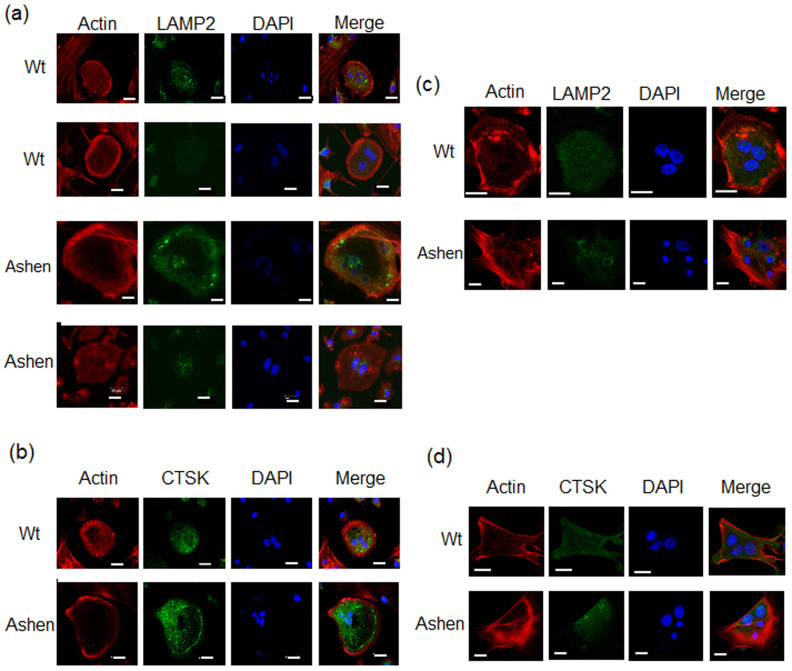
Immunofluorescence observation of actin ring formation and lysosomal proteins in OCLs from wild-type and *ashen* mice. The cells on cover-glass were fixed, permeabilized with 0.3% Tween-20 in PBS, and then allowed to react with phalloidin for F-actin (red) or antibodies for lysosomal proteins (green). After washing, the samples were incubated with a fluorescence-labeled secondary antibody and then were visualized by confocal laser microscopy. (a) On non-coated glass slip, LAMP-2 antibody. (b) On non-coated glass slip, CTSK antibody. (c) On vitronectin-coated glass slip, LAMP-2 antibody. (d) On vitronectin-coated glass slip, CTSK antibody. Bar: 50 μm.

To determine whether Rab27A affects the transport of endosomes/lysosomes in OCLs or not, we analyzed actin-ring formation and subcellular localization of LAMP2 in wild-type and *ashen* OCLs on non-coated glass slip. We observed an ovary-shape and clear form in the actin-ring formation of wild-type OCLs, whereas *ashen* OCLs displayed an irregular shape and unclear form (Fig. 6a). The subcellular distribution of LAMP2 in wild-type OCLs was seen in various punctate structures located within the actin ring (Fig. 6a). However, the staining patterns showed two different localizations of LAMP2 in *ashen* OCLs: irregular-shaped punctate structures along the actin-ring distant from the nuclei (Fig. 6a, the upper panel), and those located predominantly around the nuclei (Fig. 6a, the lower panel). We also determined the subcellular localization of CTSK in wild-type and *ashen* OCLs. In wild-type OCLs, the staining of CTSK was detected in punctate structures within the central cytoplasm around the nuclei (Fig. 6b). However, in *ashen* OCLs, the subcellular localization of CTSK was predominantly present in the area circumscribed by the peripheral actin ring, although it was detected in various punctate structures around the multiple nuclei (Fig. 6b).

To further observe on distribution of OCLs on dentin or bone slice, we performed alternative experiments on vitronectin-coated glass coverslips. Recently, Szewczyk *et al.* reported that the distribution of F-actin and lysosomal proteins in OCLs is very similar on bone slice and vitronectin-coated glass coverslips^27^. On vitronectin, *ashen* OCLs displayed more irregular shape of F-actin formation than wild-type OCLs (Fig. 6c, d), although the localizations of LAMP2 between wild-type and *ashen* OCLs were not so different (Fig. 6c, 6d). However, the localization of CTSK was exclusively around the nuclei in *ashen* OCLs, whereas that was detected in the central cytoplasm in wild-type OCLs. The results also indicate that the abnormal transport of LROs in *ashen* OCLs compared to wild-type OCLs.

### Endosomal/lysosomal proteins in ashen OCLs are perturbed

**Figure 7:**
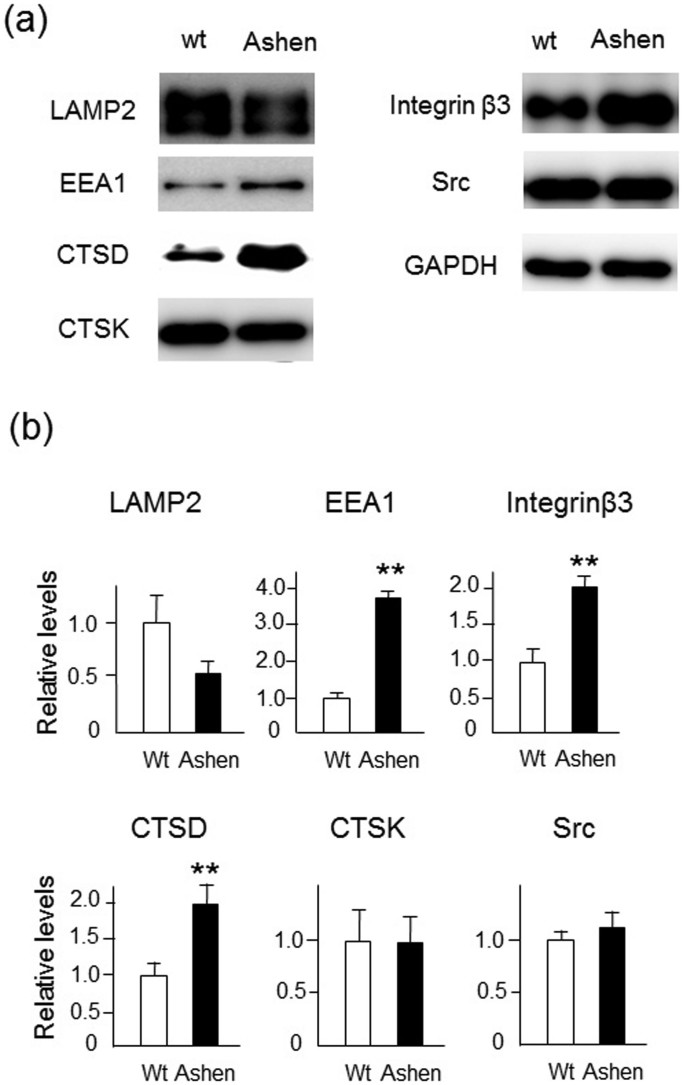
Lysosomal function in OCLs from wild-type and *ashen* mice. (a) BMMs were prepared by the method described in “Experimental Procedures”. The OCLs were further differentiated BMMs that were cultured with RANKL (50 ng/ml) and M-CSF (10 ng/ml) for 6 days. The same protein amounts of OCL-lysates were subjected to SDS-PAGE followed by western blotting with antibodies to LAMP2, EEA1, cathepsin D (CTSD), CTSK, integrin β3, Src, and GAPDH. (b) Densitometric analysis for the quantification of each protein in the cell lysate of both cell types. The relative levels were defined as the chemiluminescence intensity per mm^2^ measured by LAS4000-mini. The data are presented as the mean ± S.D. of values from four independent experiments. **P < 0.01 for the indicated comparison.

The levels of various endosomal/lysosomal proteins in OCLs were determined by western blot analysis of wild-type and *ashen* OCL cell lysates. The expression level of LAMP-2 protein in *ashen* OCLs was lower than that in wild-type OCLs, although it was not statistically significant (Fig. 7). In contrast, the EEA1 and cathepsin D, (lysosomal aspartic protease), protein levels in *ashen* OCLs were higher than those in wild-type OCLs (Fig. 7). However, CTSK expression levels between wild-type and in *ashen* OCLs were similar (Fig. 7). In addition to lysosomal proteins, we found that expression of integrin β3, (OCL marker protein present in the plasma membrane) was significantly higher in *ashen* OCLs than that in wild-type OCLs (Fig. 7). On the other hand, the levels of Src, a cytosolic OCL marker protein, between wild-type and *ashen* OCLs were similar (Fig. 7). It should be noted that the protein expression of integrin β3 in *ashen* OCLs was highly increased compared with wild-type OCLs (Fig. 7), but the mRNA level of integrin β3 in *ashen* OCLs was comparable to wild-type OCLs (Fig. 3), suggesting that transport and/or degradation rate of integrin β3 between wild-type and *ashen* OCLs may be different. Thus, the expression levels of various endosomal/lysosomal proteins were perturbed in *ashen* OCLs compared to those in wild-type OCLs.

### Impaired bone resorption activity in *ashen* OCLs

**Figure 8:**
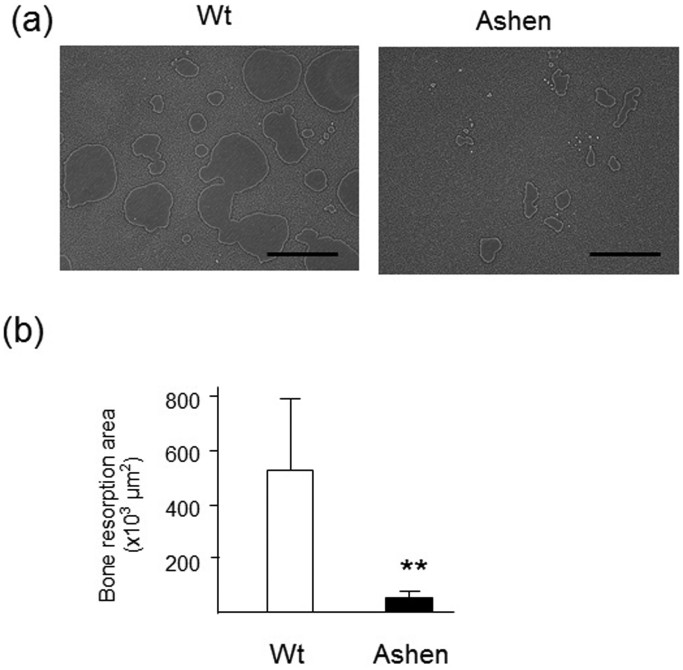
Bone resorption activity of OCLs from wild-type and *ashen* mice. (a)Photograph of the bone-resorption activity of OCLs from wild-type and *ashen* mice. (b) The bone resorption area was analyzed by using the BZ analyzer software, dynamic cell count system (Keyence). Data are presented as s the mean ± S.D. of three independent experiments. **P < 0.01 for the indicated comparisons.

Finally, the bone resorption activity of OCLs was analyzed by a pit formation assay, using OCLs derived from wild-type and *ashen* mice. After comparing the resorption pit area, *ashen* OCLs exhibited a marked reduction in the resorbing activity, while wild-type OCLs had a moderate resorbing activity (Fig. 8a, b). Taken together, we concluded that the OCLs from *ashen* mice express an abnormal lysosome function.

## Discussion

In this study, we have demonstrated the increase in Rab27A expression during OCL differentiation. Studies on siRNA knockdown in RAW-D cells or BMMs derived from *ashen* mice showed that Rab27A deficiency in OCLs is responsible for induction of multinucleation and giant cell formation. Stimulation with M-CSF or RANKL caused enhanced phosphorylation levels of Erk, p-38, and Src in *ashen* OCLs compared to wild-type OCLs. The *ashen* OCLs exhibited marked reduction in the bone resorbing activity and abnormal lysosome localization, indicating that Rab27A deficiency caused abnormal transport of LROs in the OCLs.

Rab27A-deficient OCLs were larger in size and multinucleated. This phenotype is probably due to the enhancement of several signaling pathways through cell surface receptors such as c-fms and RANK. Indeed, we observed increased phosphorylation levels of Erk, p-38 and Src signaling thorough the M-CSF c-fms complex, and Erk, p-38, and Akt signaling through the RANKL-RANK complex in Rab27A-deficient OCLs. This phenotype is reminiscent of Vps35 deficiency in OCLs^28^. Vps35 is a critical essential component of the retromer protein complex that regulates transmembrane proteins from the endosomes to the Golgi apparatus. Vps35-deficient OCLs shows a larger cell size and are multinucleated due to enhanced RANK signaling^28^. In such a case, increased cell surface levels of RANK in Vps35-deficient OCLs causes enhanced RANK signaling, resulting in larger cell size and multinucleation. However, in case of Rab27A-deficient OCLs, the surface level of RANK was comparable to that of wild-type cells. On the other hand, the cell surface level of c-fms was increased in these cells compared to wild-type OCLs. One possible explanation that normal RANK surface level with enhanced RANKL signaling in Rab27-deficient OCLs is continuous remaining of RANK on endosomes. Alternatively, abnormal transport of adaptor proteins of RANK (e.g.TRAF6) in Rab27-deficient OCLs may cause the enhanced signaling.

Rab27A-deficient OCLs shows a markedly impaired bone resorption activity. Bone resorption is a complex process combining secretion of protons by the vacuolar H+-ATPase and various lysosomal enzymes, and endocytosis/transcytosis of the degraded products via vesicle transport from apical to basolateral membrane^29^. Previous studies with various leukocytes have shown that Rab27A is involved in both exocytosis/secretion and endocytosis/phagocytosis^30^,^31^,^32^. Rab27A-deficient neutrophils^33^,^34^, eosinophils^35^, basophils^36^ all show impaired exocytosis. In contrast, receptor-mediated phagocytosis is enhanced in Rab27A-deficient macrophages. Rab27A-deficient dendritic cells show increased phagosome acidification and antigen degradation, causing a defect in antigen cross-presentation^30^. Thus, Rab27A mediated-bone resorption may be quite different mechanisms to its phagocytosis.

The LAMP2 distribution was observed in two different regions in *ashen* OCLs; the peripheral region along the actin-ring, and the perinuclear region. Similarly, CTSK was predominantly detected in the similar peripheral region in *ashen* OCLs. Contrastingly, the distribution of LAMP2 and CTSK in wild-type OCLs was seen in various punctate structures within the actin ring, which is consistent with previous results^37^. The abnormal lysosomal protein distribution and severely impaired bone resorption activity in *ashen* OCLs suggests that Rab27A probably regulates cellular distribution and secretion of LROs in OCLs.

Rab27A is speculated to play a unique role in OCLs, since its depletion results in different phenotypes compared to that expressed during deletion of other Rab proteins in OCLs. Rab3, for example, is involved in regulated exocytosis in secretory cells such as neurons and neuroendocrine cells, whose characteristics are similar to those of Rab27A^2^. Rab3D is the Rab3 isoform predominantly expressed in OCLs. Although Rab3D-deficient OCLs exhibit an abnormal ruffled border, the localization of Rab3D in the post-TGN (trans-Golgi network) vesicles and not in the OCL lysosomes, suggests that Rab3D may regulate the biogenesis of a secretory compartment in the TGN^38^. Rab7 in OCLs is thought to be implicated in the formation of ruffled borders^38^. *Rab7*-gene knockdown experiments using antisense oligo-deoxynucleotides have revealed that Rab7 is required for polarization of ruffled border formation, as well as bone resorption by OCLs via transport of V-ATPase to the ruffled border membrane^38^. Rab13 is up-regulated during differentiation of human peripheral blood monocytic cells into OCLs^39^. Since Rab13 is localized in vesicles between the trans-Golgi network and basolateral membrane domain, it is not involved in endocytosis or transcytosis of bone degradation products^39^. Rab38 is involved in sorting of protein to the melanosome in melanocytes, and phagosomal maturation in macrophages. However, OCLs derived from Rab38-deficient (*Chocolate*) mice show no phenotype, indicating redundant function(s) of other Rab protein(s)^40^. Considering the findings about other rab proteins in OCLs and our results, it is speculated that Rab27A may have redundant function of Rab38 in OCLs.

The role of Rab27A/B in osteoblasts has been reported. Suppression of Rab27A or RAb27B by RNA interference causes markedly reduced secretion of RANKL in murine osteoblastic cell line ST2 cells^41^. These results also suggest that Rab27deficiency in osteoblasts results in reduced OCL activation.

In conclusion, this study shows that Rab27A deficiency causes an abnormal transport of LROs in OCLs. Moreover, Rab27A deficiency shows the increased surface level of c-fms, and the delayed down-regulation of RANK in *ashen* cells, resulting in enhanced signaling of M-CSF and RANKL.

## Methods

### Antibodies and reagents

Macrophage colony stimulating factor (M-CSF) was purchased from Kyowa Hakko Kogyo (Tokyo, Japan). Recombinant receptor activator of nuclear factor kappa-B ligand (RANKL) was prepared as described previously^42^. Antibodies (Abs) were purchased as follows: Rabbit polyclonal anti-GM130 (Cat. NO. PM061) and, Rabbit polyclonal anti-Calnexin (Cat.No. PM060) were from MBL (Nagoya, Japan). Rat monoclonal anti-LAMP2 (Cat. No. 1921-01) were from Southern Biotechnology Inc, Birmingham, AL, USA. Mouse monoclonal anti-Src (proto-oncogene tyrosine-protein kinase) (Cat. No. 05-184, Upstate Biotechnology, Lake Placid, NY, USA). Rabbit polyclonal anti-c-fms (colony-stimulating factor 1 receptor) (Cat. No. sc-692), Rabbit polyclonal anti-RANK (receptor activator of nuclear factor kappa-B) (Cat. No. sc-9072), and mouse monoclonal anti-NFATc1 (nuclear factor of activated T-cells, cytoplasmic 1) (Cat. No. sc-7294) were from Santa Cruz Biotechnology (Santa Cruz, CA, USA). Abs specific for phospho-Erk1/2 (extracellular signal-regulated kinase) (Cat. No. 9101S, Thr202/Tyr204), phospho-Akt (protein kinase B) (Cat. No. 9271S, Ser473), phospho-p38 (Cat. No. 9211S, Thr180/Tyr182,) and phospho-Src (Cat No. 2105S, Tyr527) were from Cell Signaling Technology (Danvers, MA, USA). Rabbit polyclonal Anti-Rab27A/B from Immuno Biological Laboratories Ltd. (Takasaki, Japan). Rabbit polyclonal anti-cathepsin D or cathepsin K was prepared as described previously^43^,^44^. The Osteo Assay Plate was from Corning (Corning, NY, USA). All other reagents, including phenylmethylsulfonyl fluoride and the protease inhibitor cocktail, were from Sigma-Aldrich (Saint-Louis, MO, USA). Alexa Fluor 488 goat anti-rabbit IgG and Alexa Fluor 555 goat anti-rat IgG were from Molecular Probes/Invitrogen (Eugene, Oregon, USA).

### Animals

The *ash/ash* (C3H/He background) and control C3H/He mice described previously were originally provided by N.A. Jenkins (National Cancer Institute, Frederick, Maryland, USA)^45^. All animals were maintained according to the guidelines offset by the Japanese Pharmacological Society. All animals, their handling, and experiments were approved by the Animal Research Committee of the Graduate School of Biomedical Sciences, Nagasaki University.

### Cell culture

Isolation of BMMs was performed according to a previously described method^46^. Briefly, marrow cells were cultured from mice femurs and tibias overnight in α-minimal essential medium (α-MEM) (Wako Pure Chemicals, Osaka, Japan) containing 10% fetal bovine serum (FBS), supplemented with 100 U/ml of penicillin and 100 μg/ml of streptomycin in the presence of M-CSF (10 ng/ml) at 37 °C in 5% CO_2_. The non-adherent cells were harvested; stroma-free bone marrow cells were cultured in the presence of 10 ng/ml of M-CSF. After 4 days, the non-adherent cells were washed out, and the adherent cells were used as BMMs. The BMMs were replated, and then further cultured with new medium containing M-CSF (10 ng/ml) and RANKL (50 ng/ml) for the times indicated.

### mRNA expression profiling of OCL differentiation by Affymetrix Microarray

To identify the up-regulated genes during OCL differentiation, we utilized the Affymetrix microarray system (Affymetrix, Santa Clara, CA, USA). The data were obtained from BMMs cultured for 72 h on a plastic surface (rapid differentiation) and a dentin slice (slow differentiation) with M-CSF (30 ng/ml) and RANKL (50 μg/ml) were compared. Total RNA from the two types of BMMs were isolated with Trizol Reagent (Invitrogen, Carlsbad, USA). The samples were further purified using the RNeasy Mini Kit (QIAGEN, Tokyo, Japan) as per the manufacturer's instructions. The hybridized microarray gene chip arrays were analyzed according to the manufacturer's instructions and scanned by an Affymetrix Scanner.

### Immunofluorescence microscopy

Immunocytochemistry was performed as described previously^43^. Briefly, the cells were grown on glass cover slips and fixed with 4.0% paraformaldehyde in PBS for 30 min at room temperature. The fixed cells were then washed with 50 mM NH_4_Cl in PBS for quenching, and permeabilized with 0.2% Tween-20 in PBS for 10 min. The cells were incubated with 0.2% gelatin in PBS for 1 h, and subsequently incubated overnight at 4°C with primary antibodies. After washing, the cells were stained by second antibodies such as Alexa Fluor 488 goat anti-rabbit IgG or Alexa Fluor 594 (Cell Signaling Technology, Danvers, MA, USA) goat anti-rabbit IgG. Actin cytoskeleton staining with Alexa 594-phalloidin (Invitrogen) or nuclear staining with 4′,6-diamidino-2-phenylindole (DAPI, Invitrogen Carlsbad, CA, USA) was performed. The samples were subjected to microscopy using a laser-scanning confocal imaging system (LSM510, 710 or 780 META; Carl Zeiss, AG, Jena, Germany).

### Quantitative RT-PCR

Total RNA was isolated with Trizol Reagent, and reverse transcription was performed using oligo(dT)_15_ primer (Promega) and Revertra Ace (Toyobo, Osaka, Japan). Quantitative real-time PCR was performed using a MX3005P QPCR system (Agilent Technology, La Jolla, CA, USA). cDNA was amplified using Brilliant III Ultra-Fast SYBR QPCR Master Mix (Agilent Technology) according to the manufacturer's instructions. The following primer sets were used: GAPDH forward, AAATGGTGAAGGTCGGTGTG; GAPDH reverse, TGAAGGGGTCGTTGATGG; Rab27A forward, TTCCTGCTTCTGTTCGACCT; Rab27A reverse, GGCAGCACTGGTTTCAAAAT; Rab27B forward, GATGGAGACTATGATTATCTG; Rab27B reverse, TCCATTGACAGTGTCCGGAACC; RANK forward, CTTGGACACCTGGAATGAAGAAG; RANK reverse, AGGGCCTTGCCTGCATC; c-fms forward, TTGGACTGGCTAGGGACATC; c-fms reverse, GGTTCAGACCAAGCGAGAAG; DC-STAMP forward, CTAGCTGGCTGGACTTCATCC; DC-STAMP reverse, TCATGCTGTCTAGGAGACCTC; OC-STAMP forward, TGGGCCTCCATATGACCTCGAGTAG; OC-STAMP reverse, TCAAAGGCTTGTAAATTGGAGGAGT; CTSK forward, CAGCTTCCCCAAGATGTGAT; CTSK reverse, AGCACCAACGAGAGGAGAAA; Integrin β_3_ forward, TGTGTGCCTGGTGCTCAGA; Integrin β_3_ reverse, AGCAGGTTCTCCTTCAGGTTACA; Src forward, AGAGTGCTGAGCGACCTGTGT; Src reverse, GCAGAGATGCTGCCTTGGTT; CTR forward, CGCATCCGCTTGAATGTG; CTR reverse, TCTGTCTTTCCCCAGGAAATGA.

### Transient transfection of RAW-D cells

RAW-D cells were cultured in α-MEM supplemented with 10% FBS. All cells were maintained in a 70% confluent at 37°C in an incubator containing 5% CO_2_, and transiently transfected with pEGFP-C1-Rab27A using SuperFect™ Transfection Reagent (Quiagen, Germany) according to the manufacture's protocol.

### Flow cytometry analysis

The cell suspension (2 × 10^6^ cells/100 μl) was incubated on ice for 20 min with primary antibodies appropriately diluted with PBS containing 1% normal goat serum. Samples were preincubated with Fc Block (anti-mouse CD16/CD32 antibody) (BD Pharmigen), and subsequently with specific antibodies or control antibodies RANK (anti-mouse CD265-PE conjugated) or c-fms (anti-mouse CD115-PE conjugated) or isotype control (rat IgG2a k-PE conjugated) for 15 min on ice. Flow cytometric analyses were performed on a BD FACS Verse Cytometer.

### TRAP staining method

The cells were fixed with 4% paraformaldehyde and stained for TRAP activity using a previously described method^47^ TRAP-positive cells with 3 or more nuclei were regarded as mature osteoclasts. Murine monocytic cell line RAW-D cells, were kindly provided by Prof. Toshio Kukita (Kyushu University, Japan) and cultured in α-MEM containing 10% FBS with RANKL (50 ng/ml)^25^ were used for this experiment.

### Small interfering RNA (siRNA)

siRNA experiments in OCLs were performed as described previously^48^. The target sequence of murine Rab27A siRNA was: GGACUUAAUCAUCTAAGAGAAUGGAA (Rab27A siRNA). Briefly, RAW-D cells plated at 5 × 10^4^ cells on 60-mm plates were cultured in the presence of RANKL in antibiotic-free media. The siRNA was transfected into RAW-D cells using Lipofectamine RNAiMAX™ transfection reagent (Invitrogen, Carlsbad, CA, USA) according to the manufacturer's instructions. BLOCK-iT™ Alexa Fluor Red Fluorescent Oligo (Life Technologies, Carlsbad, CA, USA) was used to optimize the delivery of siRNA. The cells were incubated with 10 pmol of siRNA for 24 h. For TRAP staining, the cells were incubated for an additional 5 days.

### Bone resorption assay

The bone-resorbing activity of OCLs was determined using the Osteo Assay Stripwell Plate for 5 days of culture. Images for cell area, bone resorption area, and nuclei counting were taken with anal-in-one type fluorescence microscope BZ-9000 (Keyence). Deconvolution for the cell and bone resorption area was analyzed by using the BZ analyzer software, dynamic cell count system (Keyence).

### RANK degradation assays

OCLs were grown in a 3.5 cm dish, and incubated in α-MEM supplemented with 0.1% BSA lacking FBS for 2 h. Following this, they were incubated with 50 ng/ml of RANKL for the indicated period. The cells were harvested with a cell lysis buffer (50 mM Tris-HCl (pH 8.0), 1% Nonidet P-40, 0.5% sodium deoxycholate, 0.1% SDS, 150 mM NaCl, 1 mM phenylmethylsulfonyl fluoride and proteinase inhibitor cocktail).

### Western blot analysis

BMMs were stimulated with or without RANKL in the presence of M-CSF for the indicated amount of time. Cells were rinsed twice with ice-cold PBS, and lysed in a cell lysis buffer (50 mM Tris-HCl [pH 8.0], 1% Nonidet P-40, 0.5% sodium deoxycholate, 0.1% SDS, 150 mM NaCl, 1 mM PMSF, and proteinase inhibitor cocktail). The protein concentration of each sample was measured with BCA Protein Assay Reagent (Thermo Pierce, Rockford, IL, USA) according to the manufacturer's instructions. Five micrograms of lysate protein was applied to each lane. Following SDS-PAGE, proteins were electroblotted onto a polyvinylidene difluoride membrane. The blots were blocked with 3% skim milk/TBS (Tris-buffered saline)-0.1%Tween for 1 h at room temperature, probed with primary antibodies overnight at 4°C. They were then washed, and incubated with HRP-conjugated secondary antibodies. These were finally detected with ECL-prime (GE Healthcare Life Sciences, Little Chalfont, UK). The immunoreactive bands were analyzed using the LAS4000-mini (Fuji Photo Film, Tokyo, Japan).

### Statistical analysis

Quantitative data are presented as means ± standard deviation (S.D.). The Tukey-Kramer method was used to identify differences between concentrations when ANOVA indicated that a significant difference (*P < 0.05 or **P < 0.01) existed.

## Supplementary Material

Supplementary Information Supplementary Information
