# Supplementary material for: Rab27A Regulates Transport of Cell Surface Receptors Modulating Multinucleation and Lysosome-Related Organelles in Osteoclasts
Source: Sci Rep. 2015 Apr 16;5:9620. doi: 10.1038/srep09620 (PMC5381753; doi:10.1038/srep09620)
Supplement: Supplementary Information [file srep09620-s1.pdf]

Supplementary Information

Rab27A Regulates Transport of Cell Surface Receptors Modulating Multinucleation and Lysosome-Related Organelles in Osteoclasts

Megumi Shimada-Sugawara[1, 2], Eiko Sakai[1], Kuniaki Okamoto[1], Mitsunori Fukuda[3], Tetsuro Izumi[4], Noriaki Yoshida[2], and Takayuki Tsukuba[1]*

From the [1]Division of Dental Pharmacology, [2]Division of Orthodontics and Dentofacial Orthopedics, Graduate School of Biomedical Sciences, Nagasaki University, Nagasaki 852-8588, Japan, [3]Laboratory of Membrane Trafficking Mechanisms, Department of Developmental Biology and Neurosciences, Graduate School of Life Sciences, Tohoku University, Aobayama, Aoba-ku, Sendai, Miyagi 980-8578, Japan; [4]Department of Molecular Medicine, Institute for Molecular and Cellular Regulation, Gunma University, Maebashi 371-8512, Japan.

Contents

Supplementary Figures

Figure S1: Up-regulation of Rab27A during osteoclastogenesis……………………………….3
Figure S2: Subcellular localization of GFP-Rab27A expressed RAW-D cells………………4
Figure S3: Original gel images of immunoblot analysis……………………………………5
Figure S4: Original gel images of immunoblot analysis……………………………………6
Figure legends: ………………………………………………………………………..………7

(a)

Gene title	Log ratio
calcitonin receptor	3.84
cathepsin K	3.29
transmembrane 7 superfamily member 4 (DC-STAMP)	3.14
carbonic anhydrase 2	3.47
acid phosphatase 5, tartrate resistant (TRAP)	2.41
Rab27A	1.26
Rab27B	-1.09

(b)

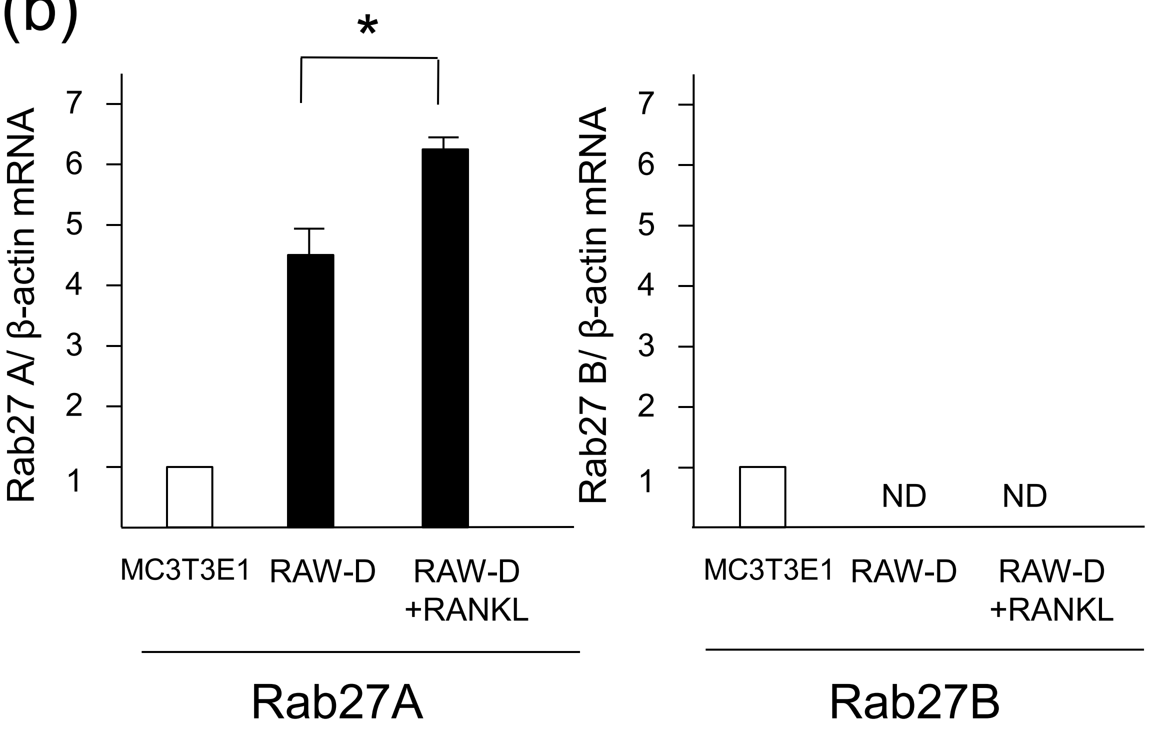

Supplementary Figure 1

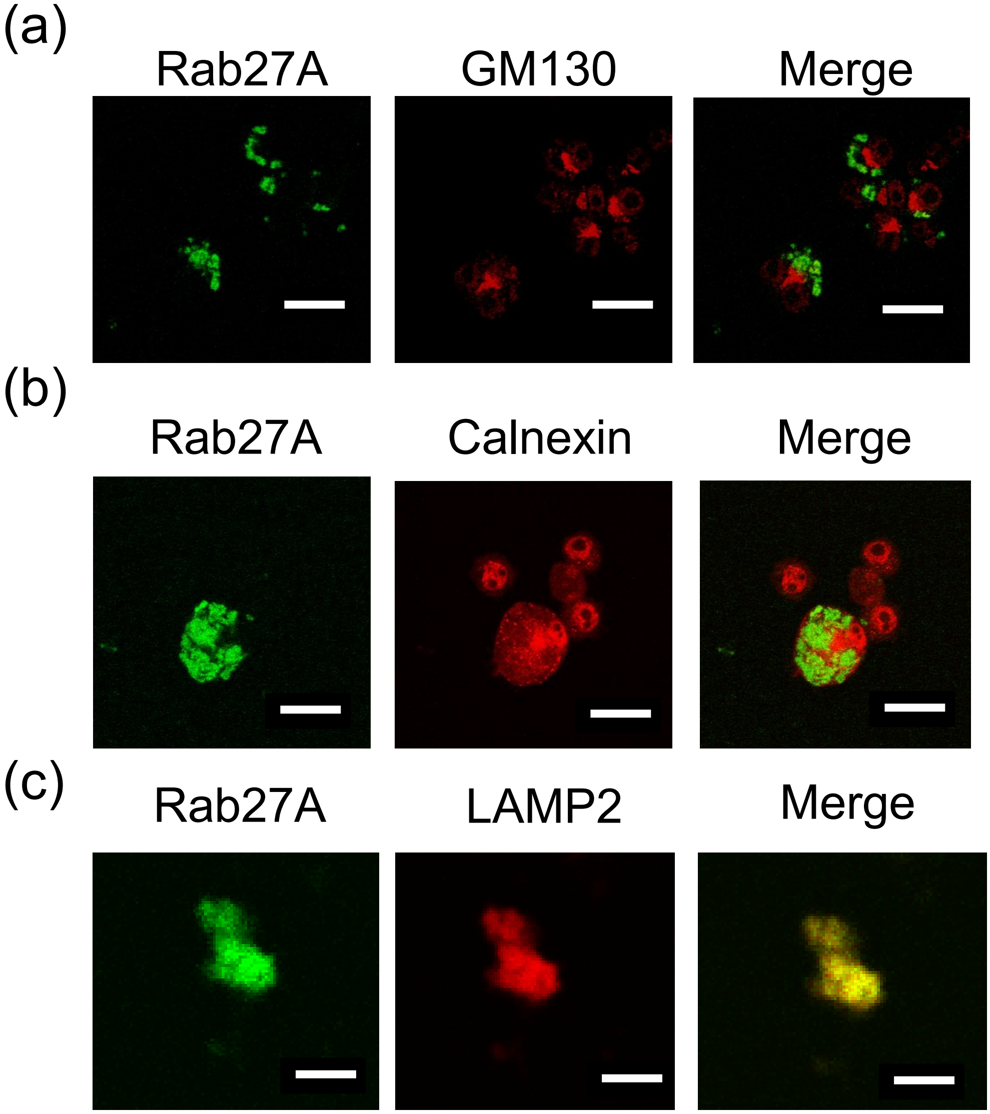

Supplementary Figure 2

Fig. 4(a)

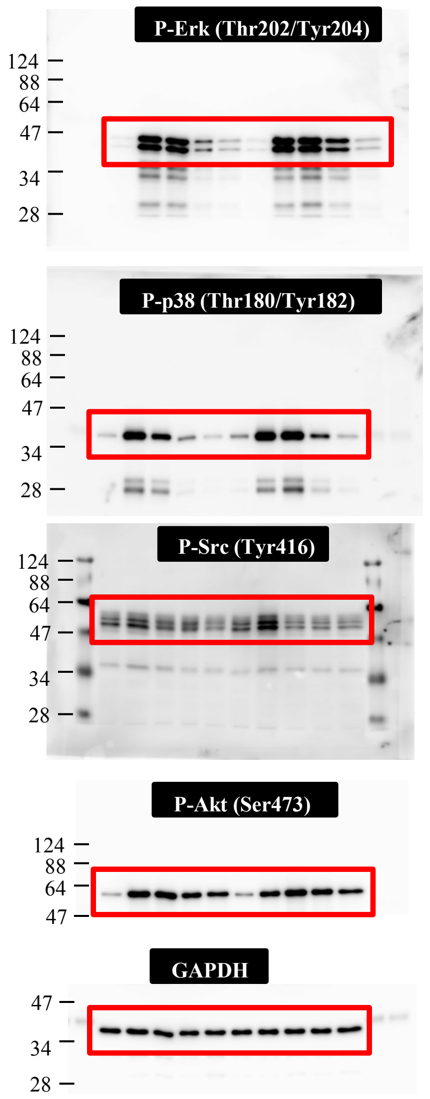

Fig. 4(b)

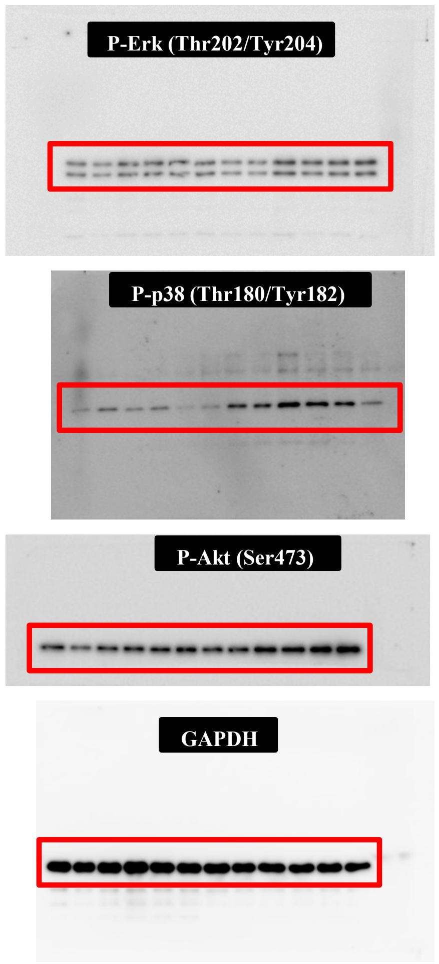

Fig. 5(g)

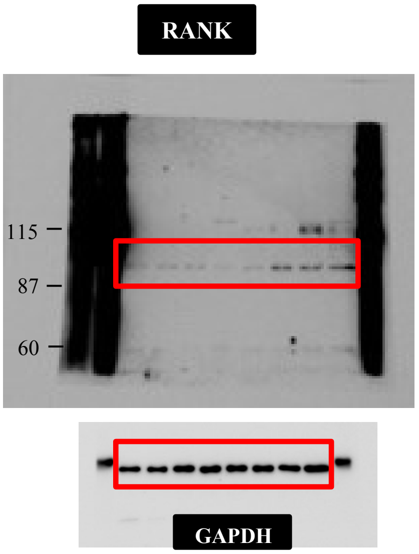

Supplementary Figure 3

Fig. 5(a)

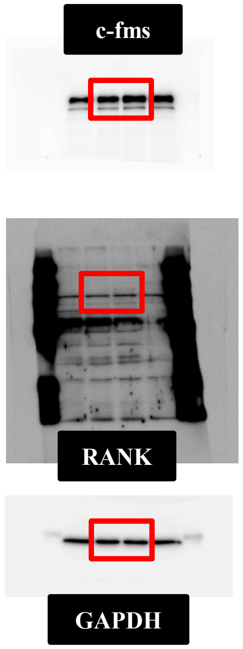

Fig. 5(c)

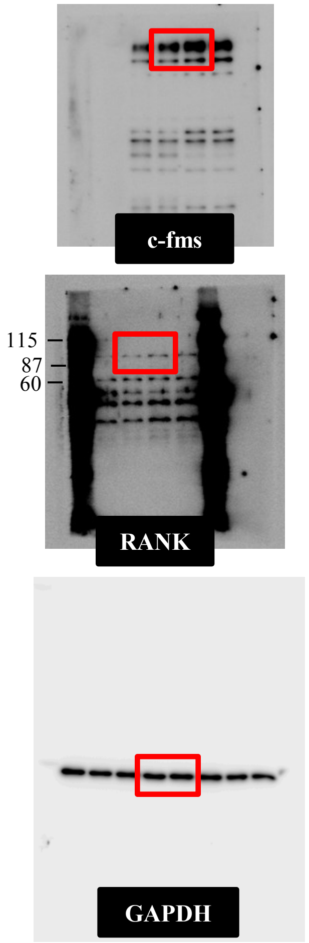

Fig. 7(a)

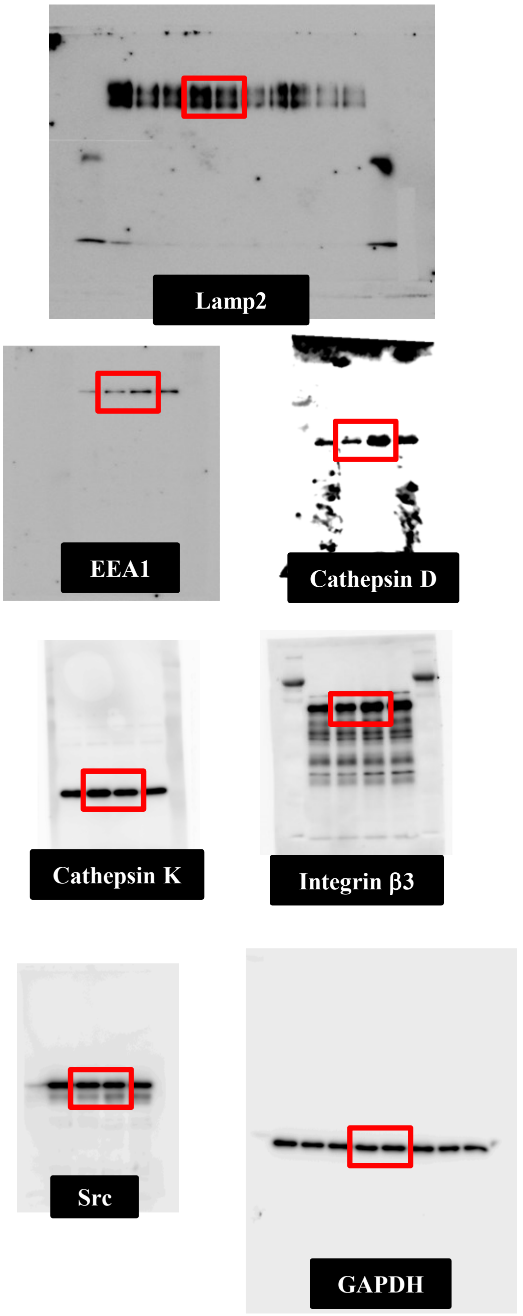

Supplementary Figure 4

Supplementary Figure 1. Up-regulation of Rab27A during osteoclastogenesis. (a) List of up-regulated transcripts in rapid differentiating OCLs compared to slow differentiating OCLs. BMMs were cultured on plastic plate (rapid differentiating conditions) and dentin (slow differentiating conditions) for 72 h in the presence of M-CSF (30ng/mL) and RANKL (50ng/mL). Total RNA from these cells was analyzed by *Affymetrix Microarray* system. (b) Quantitative RT-PCR determination of mRNA expression levels of Rab27A and Rab27B in MC3T3, RAW-D, and RANKL-stimulated RAW-D cells (OCLs). The data are represented as mean ± Standard Deviation (S.D.) of values from three independent experiments. $^*P < 0.05$ for the indicated comparisons.

Supplementary Figure 2 Subcellular localization of GFP-Rab27A expressed RAW-D cells. The cells on glass cover-slips were fixed, permeabilized with 0.2% Tween-20 in PBS, and then allowed to react with (a) anti-GM130 (marker for the Golgi), (b) anti-calnexin (marker for ER), (c) anti-LAMP2 (marker for late endosomes/lysosomes) antibodies on the glass cover slips. After being washed, the samples were incubated with a fluorescence-labeled secondary antibody and then were visualized by confocal laser microscopy. Bar: 10 μm
